# Measurement of the Acoustic Non-Linearity Parameter of Materials by Exciting Reversed-Phase Rayleigh Waves in Opposite Directions

**DOI:** 10.3390/s20071955

**Published:** 2020-03-31

**Authors:** Bingsheng Yan, Yuzhou Song, Shijie Nie, Mingchao Yang, Ziran Liu

**Affiliations:** School of Mechanical and Electrical Engineering, Henan University of Technology, Zhengzhou 450001, China

**Keywords:** nonlinear ultrasonics, Rayleigh waves, acoustic non-linearity parameter, FEM simulation, crack detection

## Abstract

The acoustic non-linearity parameter of Rayleigh waves can be used to detect various defects (such as dislocation and micro-cracks) on material surfaces of thick-plate structures; however, it is generally low and likely to be masked by noise. Moreover, conventional methods used with non-linear Rayleigh waves exhibit a low detection efficiency. To tackle these problems, a method of exciting reversed-phase Rayleigh waves in opposite directions is proposed to measure the acoustic non-linearity parameter of materials. For that, two angle beam wedge transducers were placed at the two ends of the upper surface of a specimen to excite two Rayleigh waves of opposite phases, while a normal transducer was installed in the middle of the upper surface to receive them. By taking specimens of 0Cr17Ni4Cu4Nb martensitic stainless steel subjected to fatigue damage as an example, a finite element simulation model was established to test the proposed method of measuring the acoustic non-linearity parameter. The simulation results show that the amplitude of fundamentals is significantly reduced due to offset, while that of second harmonics greatly increases due to superposition because of the opposite phases of the excited signals, and the acoustic non-linearity parameter thus increases. The experimental research on fatigue damage specimens was carried out using this method. The test result was consistent with the simulation result. Thus, the method of exciting reversed-phase Rayleigh waves in opposite directions can remarkably increase the acoustic non-linearity parameter. Additionally, synchronous excitation with double-angle beam wedge transducers can double the detection efficiency.

## 1. Introduction

Recent theories and related experimental studies have shown that the fatigue damage of metal materials in the early stage is related to the non-linear effect of ultrasonic waves [1,2,3,4,5]. In the early stage of fatigue damage to metals, the waveform is distorted when ultrasonic waves at a single frequency propagate therein due to the presence of various micro-defects such as dislocations, persistent slip bands (PSB), and micro-cracks, thus generating second harmonics. By using the second harmonic generation (SHG), a specific nonlinear ultrasound (NLU) technique, the fundamental and second harmonic components in the propagating wave are detected to determine the acoustic non-linearity parameter (*β*), which enables effective non-destructive testing (NDT) and non-destructive evaluation (NDE) of the degree of fatigue damage to materials and structures in the early stage [6].

Compared with body waves, Rayleigh waves show unique advantages. They can propagate on smooth curved surfaces without generating reflections, and it is feasible to test parts of the unusual shape; moreover, the energy of Rayleigh acoustic waves is concentrated on the structure surface, so it is possible to excite and collect ultrasonic waves only from one side of the structure. The measurement process is simple and easy and allows the user to judge whether defects originate from the surface or the interior of a specimen; the degree of attenuation of Rayleigh waves is less significant than body waves, and Rayleigh waves can therefore propagate further [7]. Thus, Rayleigh waves are suitable for ultrasonic testing of large-sized thick-plate complex structure; moreover, developing non-linear Rayleigh waves to test the early fatigue damage of thick-plate structures of metal materials has practical engineering significance.

Second harmonics, with a small amplitude, are likely to be masked by interference signals owing to their being affected by various factors (such as the inherent non-linearity of materials, non-linearity of the measurement system, diffraction, and attenuation) during testing [8]. Therefore, how to establish a measurement system for acquiring *β* values and developing associated signal processing methods has been the focus of much research. Jin-Yeon Kim et al. conducted non-linear ultrasonic testing of the fatigue damage of nickel-based alloy by employing PZT transducers, and optimized the test system by introducing calibration technology [9]. David Torello et al. proposed a method based on a nonlinear least squares curve-fitting algorithm for performing diffraction and attenuation correction on *β*; the measurement result indicated that the technology can improve the measurement accuracy of *β* value [10]. Sebastian Thiele et al. received ultrasonic signals by applying a non-contact, air-coupled ultrasonic transducer, which decreased the non-linear interference caused by coupling between the sensor and the test piece; they fit the measured value of *β* by using the least squares method, thus enhancing the measurement accuracy [11]. Guoshuang Shui et al. measured the ultrasonic *β* of materials by directly exciting Rayleigh waves through line contact, which provides a referential NDT method for assessment using non-linear ultrasonic waves [12].

When detecting the fatigue damage to large-area thick-plate parts by applying non-linear Rayleigh waves, *β* is low, thus causing a low detection efficiency. To solve this problem, a method of exciting reversed-phase Rayleigh waves in opposite directions is proposed to measure *β*. Gas turbine blades are subject to alternating loads for a long time during operation, which makes them prone to sudden fracture due to fatigue. 0Cr17Ni4Cu4Nb martensitic stainless steel is the raw material for gas turbine blades, which has high strength, hardness, and corrosion resistance. By taking specimens of 0Cr17Ni4Cu4Nb martensitic stainless steel subjected to fatigue damage as an example, a finite element simulation model for testing the proposed method was constructed. Moreover, the method was used to measure specimens of 0Cr17Ni4Cu4Nb martensitic stainless steel subjected to fatigue damage. The results show that, compared with conventional methods using non-linear Rayleigh waves, the fundamental amplitude is significantly reduced due to offset effects, while the second harmonic amplitude increases due to superposition because of the reversed phases of the excited signals, and *β* thus increases. Additionally, the synchronous excitation of two angle beam wedge transducers can double the detection efficiency. Thus, this method further provides a new means for rapid and accurate detection of the fatigue damage of materials.

The principle of the method for exciting reversed-phase Rayleigh waves in opposite directions is introduced in Section 2. Numerical simulation on excitation of reversed-phase Rayleigh waves in opposite directions is described in Section 3. The experimental study is discussed in Section 4. Section 5 presents the conclusions of this work.

## 2. Principle of the Method for Exciting Reversed-Phase Rayleigh Waves in Opposite Directions

### 2.1. Non-linear Characteristics of Rayleigh Waves

Generally, solid materials will lead to a non-linear effect of ultrasonic waves due to the presence of crystal structures and micro-defects therein. The ultrasonic waves at a single frequency will generate a non-linear interaction with the micro-defects in materials when propagating in solids, further generating higher harmonics. Consider a sinusoidal longitudinal wave, whose angular frequency is ω, which propagates in the x-direction through a quadratic nonlinear, isotropic, elastic material. Combining the constitutive equation for quadratic nonlinearity and the Equation of motion, one can derive the one-dimensional nonlinear wave Equation as [13]:

(1) $$\frac{\partial^{2}u}{\partial t^{2}}=c_{l}^{2}\left[1-\beta \frac{\partial u}{\partial x}\right]\frac{\partial^{2}u}{\partial x^{2}}$$

where *c_l_* is the longitudinal wave speed in the material, *β* is the acoustic non-linearity parameter, and *u* is the particle displacement. The solution to this one-dimensional wave equation is given by [13]:

(2) $$\begin{array}{ll} u(x,t) & =A_{1}\sin(kx-\omega t)-\frac{\beta k^{2}xA_{1}{}^{2}}{8}\cos[2(kx-\omega t)]+\cdots \\ & =A_{1}\sin(kx-\omega t)+A_{2}\cos[2(kx-\omega t)]+\cdots \end{array}$$

which shows the generation of the second harmonic (i.e., the wave at 2ω) [13]. Because the coefficients of higher harmonics are smaller, only the fundamental and second harmonic coefficients are considered. Here, *x* is the effective propagation distance, *t* is time, *k* is the number of ultrasonic waves, *ω* is the angular frequency, *A*_1_ and *A*_2_, refer to fundamental and second harmonic amplitudes, respectively. The acoustic non-linearity parameter is

(3) $$\beta =\frac{8|A_{2}|}{xk^{2}A_{1}{}^{2}}$$

In Equation (3), on the condition of having fixed *k* and *x*, *β* can be determined by extracting the values of *A*_1_ and *A*_2_. 

Since Rayleigh waves are generated by superposing non-uniform plane longitudinal waves with non-uniform plane shear waves at the same propagation velocity, the displacement of Rayleigh waves can be decomposed into longitudinal and horizontal components. Due to the symmetry of the third-order elastic constants of isotropic materials, only longitudinal waves are correlated with higher-order Rayleigh waves. Thus, the effect of transverse waves on surface waves can be ignored during any test using non-linear Rayleigh waves. We Fourier-transformed the received signal to obtain the amplitude of the fundamental wave and the amplitude of the second harmonic, and then used Equation (3) to determine *β*. The test method and characteristic parameters for characterizing the degradation of mechanical properties of materials of Rayleigh waves are the same as those of longitudinal waves [14]. During this investigation, the *β* value was controlled by changing the value of *A*_2_/*A*_1_^2^. 

### 2.2. Principle of Exciting Reversed-Phase Rayleigh Waves in Opposite Directions

In Equation (3), *β* is only related to the fundamental amplitude *A*_1_ and second harmonic amplitude *A*_2_ in the case of having fixed *k* and *x*. The measured fundamental amplitude is much greater than that of second harmonics during finite element simulation and testing. Second harmonics are hard to extract and the error therein is large; moreover, the calculated *β* is also low. Thus, *β* can greatly increase if the amplitude of the fundamentals is decreased, while that of second harmonics increases. A method of exciting reversed-phase Rayleigh waves in opposite directions is thus proposed. According to Equation (2), when exciting sine wave signals with phase angle *α*,

(4) $$u_{1}(x,t)=A_{1}\sin(kx-\omega t+\alpha )+A_{2}\cos[2(kx-\omega t+\alpha )]+\cdots$$

where *α* is the initial phase angle. When exciting sine wave signals with phase angle (*α* + *π*), then

(5) $$u_{2}(x,t)=A_{1}\sin(kx-\omega t+\alpha +\pi )+A_{2}\cos[2(kx-\omega t+\alpha +\pi )]+\cdots$$

When synchronously exciting two sine wave signals with the same period and amplitude while separately having phase angles α and (*α* + *π*), then

(6) $$\begin{array}{lll} u(x,t) & = & u_{1}(x,t)+u_{2}(x,t)\\ & = & A_{1}\sin(kx-\omega t+\alpha )+A_{2}\cos[2(kx-\omega t+\alpha )]\\ & & +A_{1}\sin(kx-\omega t+\alpha +\pi )+A_{2}\cos[2(kx-\omega t+\alpha +\pi )]+\cdots \\ & = & 2A_{2}\cos[2(kx-\omega t+\alpha )]+\cdots \end{array}$$

It can be seen from Equation (6) that when synchronously exciting sine wave signals with a phase difference of *π*, the fundamental amplitudes are interactively cancelled, while second harmonic amplitudes are superimposed (i.e., increased to 2*A*_2_). In practical testing, the fundamental amplitudes cannot be completely offset, and the second harmonic amplitudes are not absolutely doubled due to the presence of various influencing factors including limitation of experimental conditions, non-uniform distribution of micro-defects, and leakage of signal energy; however, the fundamental amplitude still decreases due to partial offset and the second harmonic amplitude increases due to superposition, thus significantly improving *β*.

## 3. Numerical Simulation on Excitation of Reversed-Phase Rayleigh Waves in Opposite Directions

### 3.1. Finite Element Simulation Model

**Figure 1:**
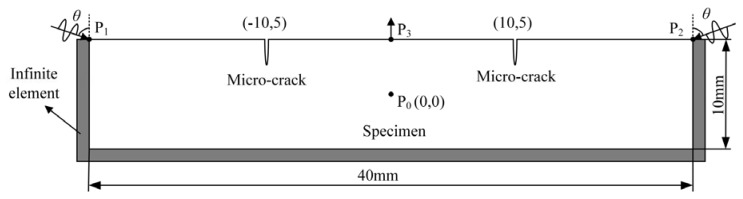
Finite element simulation model for testing the method of exciting reversed-phase Rayleigh waves in opposite directions to measure *β*.

Micro-defects (e.g., dislocation, PSB, and micro-cracks) in metal materials can all result in the change of *β*. By taking a specimen of 0Cr17Ni4Cu4Nb martensitic stainless steel with a micro-crack as an example, a finite element simulation model for testing the method of exciting reversed-phase Rayleigh waves in opposite directions was established utilizing the commercial finite element software ABAQUS, as shown in Figure 1. To reduce the computational burden, the specimen was simplified into a two-dimensional plane model with a length of 40 mm and a thickness of 10 mm, using CPS4R elements. Quadrilateral plane stress infinite elements CINPS4 were separately set at the left, right, and lower boundaries of the model to avoid reflection of ultrasonic waves at the boundaries. The size of grids (0.1 mm) in plane and infinite elements both accounted for 1/10 of the length of Rayleigh waves (*λ*_R_ = 1.18 mm). In Figure 1, the density (*ρ* = 7780 kg/m^3^), elastic modulus (*E* = 213 GPa), Poisson’s ratio (*υ* = 0.27), and Rayleigh wave speed (*C*_R_ = 2966 m/s) of the material were determined and consistent with 0Cr17Ni4Cu4Nb material parameters. A semi-elliptical micro-crack was separately set at the left and right sides of the upper surface of the specimen. Sinusoidal pulse signals with reversed-phase (*α*_1_ = 0° and *α*_2_ = 180°, respectively), with the same frequency (*f* = 2.0 MHz), amplitude (*A* = 1 μm), and number of cycles (n = 150), and with the Hanning window were synchronously excited from the two ends (P_1_ and P_2_, separated by 40 mm) on the upper surface of the model at the same incident angle (*θ* = 72°), and then signals were received at the middle of the upper surface (P_3_). The sampling frequency (*f*_s_ = 200 MHz) is consistent with the test in Section 4. 

### 3.2. Analysis of Simulation Results

In Figure 1, the coordinates of the origin point (P_0_) were (0, 0). The coordinates of the two signal excitation points (P_1_ and P_2_) were (−20, 5) and (20, 5), respectively, and the coordinates of the signal reception point (P_3_) were (0, 5). It was supposed that the micro-cracks on the surface of the specimens presented the same size and symmetric location, that is, micro-cracks with the width of 20 nm and the length of 150 μm were separately set at two locations, (−10, 5) and (10, 5), on the upper surface of the specimens for purposes of simulation.

**Figure 2:**
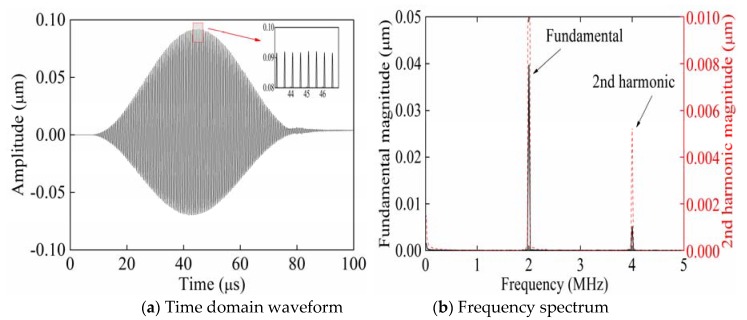
Time domain waveform and frequency spectrum of the received signals when exciting Rayleigh waves with a phase angle of 0° from P_1_.

**Figure 3:**
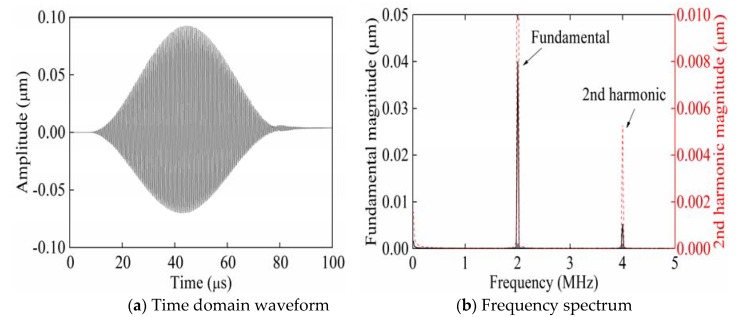
Time domain waveform and frequency spectrum of the received signals when exciting Rayleigh waves with a phase angle of 180° from P_2_.

**Figure 4:**
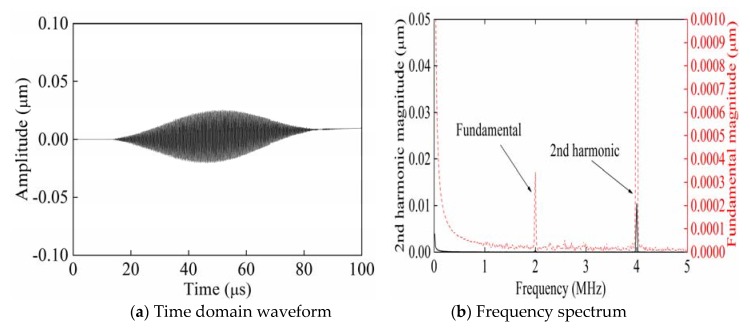
Time domain waveforms and frequency spectra of the received signals when synchronously separately exciting Rayleigh waves with phase angles of 0°and 180° from P_1_ and P_2_.

Figure 2 shows the time domain waveform and frequency spectrum of signals collected from P_3_ when exciting Rayleigh waves with a phase angle of 0° from P_1_. The partial enlarged figure shown in Figure 2a shows that the time-domain waveform of the received signal is significantly distorted compared with the time-domain waveform of the single-frequency transmitted signal. Therefore, it is verified that in Figure 2b, compared with the frequency spectrum of the single-frequency transmitted signal, the frequency spectrum of the received signal generates higher harmonics. According to frequency spectra (Figure 2, Figure 3 and Figure 4), the black solid line and red dotted line separately correspond to the left and right ordinates. In order to facilitate the observation of the smaller second harmonic amplitude or fundamental amplitude, the red dotted line is the enlarged black solid line. Figure 3 shows the time domain waveform and frequency spectrum of signals collected from P_3_ when exciting Rayleigh waves with a phase angle of 180° from P_2_. Figure 4 shows the time domain waveform and frequency spectrum of signals collected from P_3_ when synchronously exciting Rayleigh waves with phase angles of 0° and 180° from P_1_ and P_2_.

As shown in Figure 4, only superimposed second harmonic amplitudes remained in the signal, while fundamental amplitudes were cancelled due to reversed phases in the time domain waveforms when the defects were set at the left and right ends in a completely symmetric manner. The second harmonic amplitude (1.047 × 10^−2^ μm) in the spectrum approximated to the sum (1.054 × 10^−2^ μm) of second harmonic amplitudes in spectra shown in Figure 2 and Figure 3. The results show that it was feasible to measure *β* by applying the method of exciting reversed-phase Rayleigh waves in opposite directions; however, the size and location of surface micro-cracks were uncertain in practical specimens subjected to fatigue damage. Hence, a simulation was carried out using micro-cracks with asymmetric sizes and locations (Table 1).

By analyzing Table 1, it was deemed effective to measure *β* by exciting reversed-phase Rayleigh waves in opposite directions no matter whether micro-cracks were of the same size or symmetric in terms of their location in practical conditions. Although the fundamental amplitude could not be completely cancelled, it was substantially reduced; a majority of the second harmonic amplitudes increased due to superposition. As a result, *β* increased to a significant extent.

## 4. Experimental Study

### 4.1. Specimens

**Figure 5:**
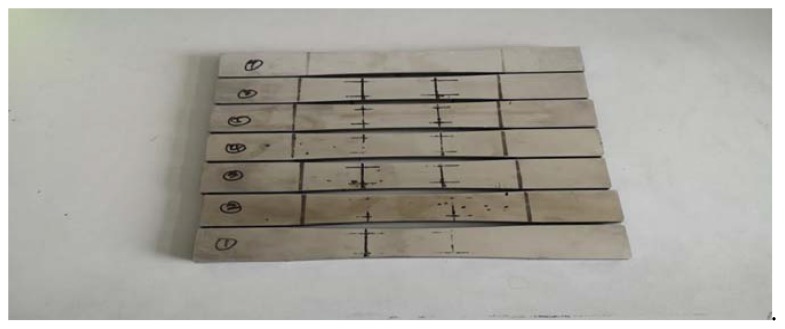
Test specimens.

**Figure 6:**
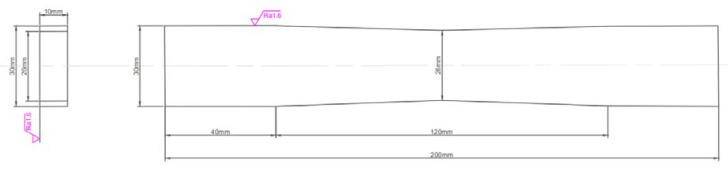
Dimensions of the test specimens.

0Cr17Ni4Cu4Nb martensitic stainless steel, the raw material used to produce gas turbine blades in power plants, was used in this experiment. As an unstable austenite structure, 0Cr17Ni4Cu4Nb was transformed into hardened martensitic stainless steel after undergoing solid treatment and over-aging treatment [15]. Figure 5 illustrates the specimens prepared by using 0Cr17Ni4Cu4Nb martensitic stainless steel. The surfaces of all specimens were ground and polished. The three-point bending fatigue test was conducted by employing a GPS2000 high-frequency (100 Hz) fatigue testing machine to prepare seven specimens with fatigue damage at 0, 6 × 10^4^, 1.2 × 10^5^, 1.8 × 10^5^, 3.0 × 10^5^, 3.6 × 10^5^, and 4.2 × 10^5^ cycles. The stress ratio (R = 0.2) and the loading frequency (*f*_l_ = 80 Hz) were determined. The dimensions of the specimens are shown in Figure 6.

### 4.2. Testing System

**Figure 7:**
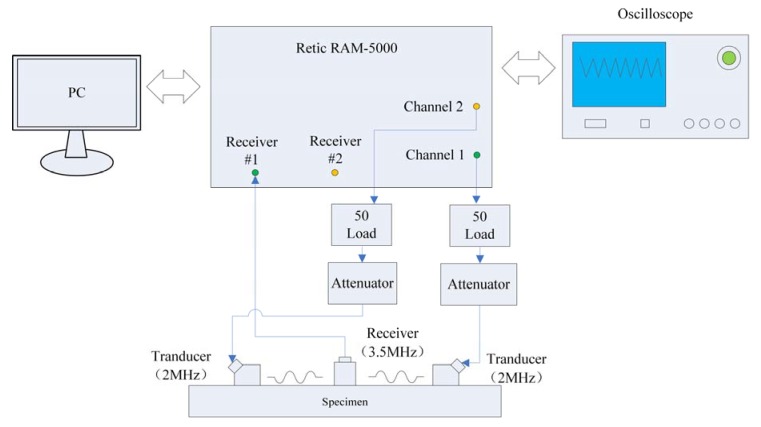
Schematic diagram of the testing system.

Figure 7 shows the testing system for measuring *β* by exciting reversed-phase Rayleigh waves in opposite directions. Two pulsed sine wave signals with a phase difference of 180°, frequency of 2.0 MHz, and periodic number of 150 were excited by using a RAM-5000-SNAP ultrasonic testing system. The pulsed sine wave signals with a phase angle of 0° drove the wedge transducer to produce Rayleigh waves at the left-hand end at a center frequency of 2 MHz. A wedge angle of 72° was used after passing through the RT-50 resistor and the attenuator to input Rayleigh waves at a frequency of 2 MHz onto the surface of the specimens; the pulsed sine wave signals with a phase angle of 180° drove the wedge transducer to produce Rayleigh waves at the right-hand end with the same parameters after propagating through the other set of identical devices to input Rayleigh waves at a frequency of 2 MHz onto the surface of the specimens; the distance between the transducers was 40 mm. The echo signals containing the second harmonic waves at the frequency of 4 MHz were received by the normal transducer for longitudinal waves at a center frequency of 3.5 MHz (Olympus, A182S-RM) in the middle of the specimens; thereafter, the signals were stored on an oscilloscope; the sample frequency was 200 MHz. Finally, the time and frequency domains of signals were analysed using the software system on a computer.

**Figure 8:**
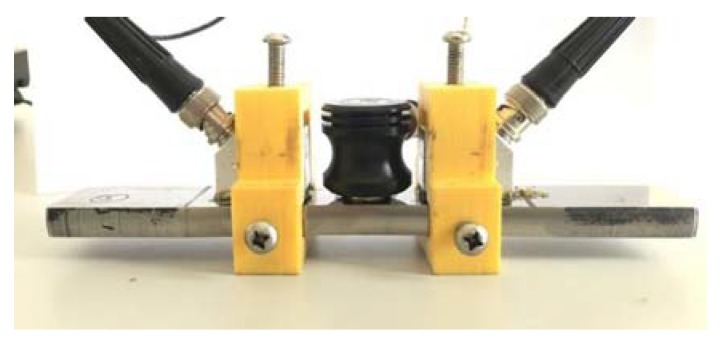
Typical specimen and installed transducers.

According to the dimensions of the wedge transducers and the specimen, a pair of fixtures was made by using polylactic acid (PLA) materials and 3D printing. A threaded hole was separately set on the top and two sides of the fixtures, so the wedge transducer could be positioned and fixed using three screws. During the experiment, the wedge transducer used to generate Rayleigh waves was fixed onto the upper surface of the specimens, and a non-volatile lithium grease was used as the coupling agent. A typical specimen with the installed wedge transducer is shown in Figure 8.

### 4.3. Experimental Results and Analysis

The fatigue-damaged specimens were tested by applying the aforementioned testing system. To reduce the boundary effect of the specimens, the effective testing length on the surface of the specimens was 40 mm. The sine wave signals with a phase angle of 0° were excited on Channel 1, and those with a phase angle of 180° from Channel 2. The normal transducer for longitudinal waves was installed in the middle of the upper surface of the specimens to receive the signals. The specimen with 1.8 × 10^5^ fatigue cycles was taken as an example for later analysis.

**Figure 9:**
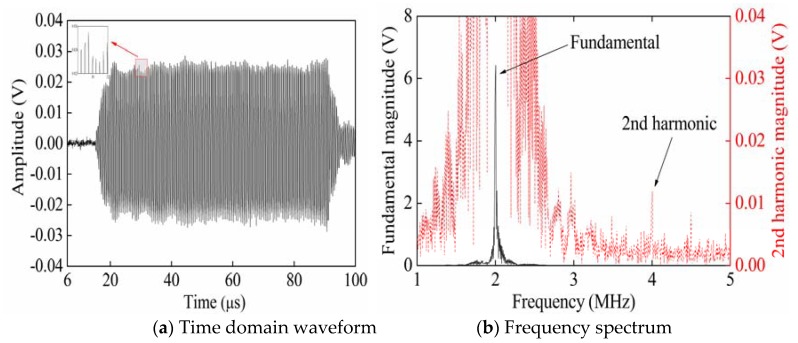
Time domain waveform and frequency spectrum of the received signals when only opening Channel 1.

**Figure 10:**
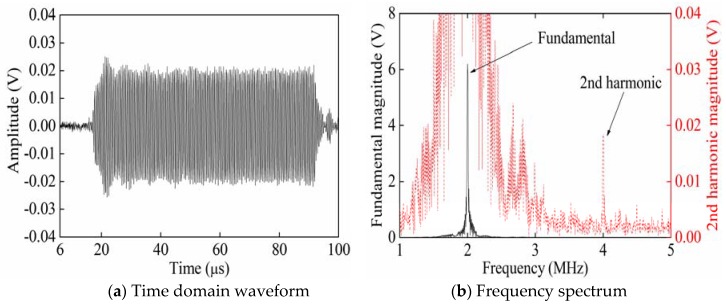
Time domain waveform and frequency spectrum of the received signals when only opening Channel 2.

**Figure 11:**
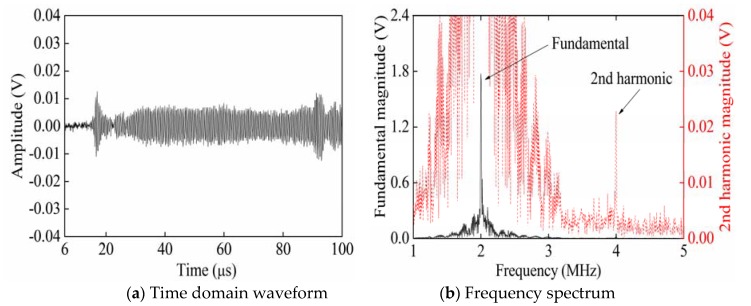
Time domain waveform and frequency spectrum of the received signals when synchronously opening Channels 1 and 2.

Figure 9 and Figure 10 show the time domain waveforms and frequency spectra of the received signals when separately opening Channels 1 and 2. The partial enlarged figure in Figure 9a shows that the time-domain waveform of the received signal is significantly distorted compared with the time-domain waveform of the single-frequency transmitted signal. Therefore, it is verified that in Figure 9b, compared with the frequency spectrum of the single-frequency transmitted signal, the frequency spectrum of the received signal generates higher harmonics. Figure 11 shows the time domain waveform and frequency spectrum of the received signals when synchronously opening Channels 1 and 2. In Figure 9b, Figure 10b, and Figure 11b, the black solid and red dotted lines separately correspond to the ordinates on the left and right-hand sides. In order to facilitate the observation of the smaller second harmonic amplitude, the red dotted line is the enlarged black solid line.

As shown in Figure 9, Figure 10 and Figure 11, the amplitude of the received signals when opening a single channel was higher than that when synchronously opening two channels, according to the time domain waveforms; by observing the frequency spectra, it can be seen that the second harmonic amplitudes of the received signals when opening a single channel were lower than that when synchronously opening two channels. The reason for this was that during synchronous excitation, the fundamental amplitude was cancelled, while second harmonic amplitude was superimposed due to reversed phases of the exciting signals. For the specimen subjected to 1.8 × 10^5^ fatigue cycles, the fundamental amplitude and the second harmonic amplitude in frequency spectra were both subjected to multiple repeated measurements to calculate the means thereof. Furthermore, *β* was calculated (Table 2).

It can be seen from the experimental result that, relative to opening a single channel, the fundamental amplitude decreased due to partial offset effect of fundamental waves when synchronously opening the two channels; the second harmonic amplitude (2.28 × 10^−2^ V) when synchronously opening two channels approximated to the sum (3.06 × 10^−2^ V) of the second harmonic amplitudes when separately opening a single channel. The experimental results are similar to the simulated results, which indicates efficacy to measure *β* by exciting reversed-phase Rayleigh waves in opposite directions.

**Figure 12:**
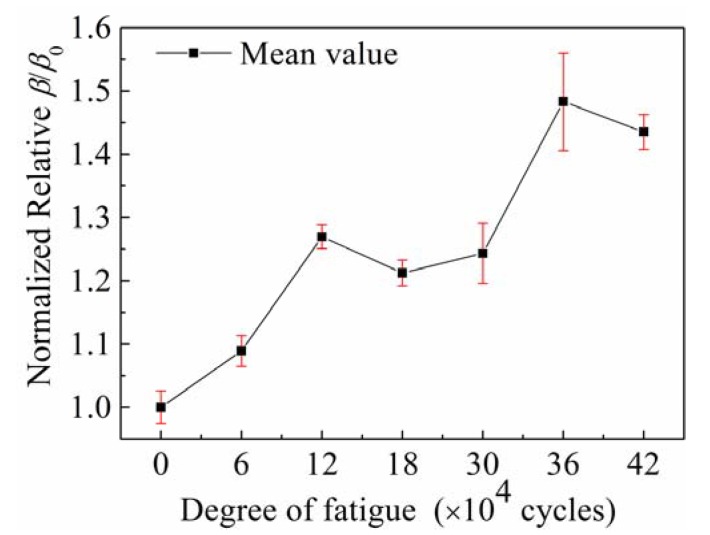
Relationship between the normalized relative *β*/*β*_0_ of the fatigue specimens and degree of fatigue.

Furthermore, *β* of the specimens with different fatigue degrees was measured by exciting reversed-phase Rayleigh waves in opposite directions. For convenience of comparison, *β*_0_ of the specimen without fatigue damages was first measured; then, *β* of the specimens subject to different numbers of fatigue cycles was measured. Afterwards, *β* was normalized according to *β*/*β*_0_. The relationship between the normalized relative *β*/*β*_0_ of the fatigue specimens and degree of fatigue is shown in Figure 12.

Figure 12 shows that although the normalized relative *β*/*β*_0_ of the fatigue specimens did not monotonically increase, it generally increased with the number of fatigue load cycles. This indicated that *β* of non-linear Rayleigh waves was sensitive to the micro-defects in the specimens induced by fatigue damage. This has potential engineering application value for the detection of surface micro-defects and prediction of the fatigue life of thick-plate structures. The proposed method of exciting reversed-phase Rayleigh waves in opposite directions provides a new technical means for developing NDT technology using non-linear ultrasonic waves.

During the experiment, the fundamental amplitudes and second harmonic amplitudes of specimens subjected to different degrees of fatigue were assessed by off-line detection, and the acoustic *β* was calculated on this basis. The difference between specimens was the main reason for the high dispersity of data observed.

## 5. Conclusions

The method of exciting reversed-phase Rayleigh waves in opposite directions is proposed to measure *β* of certain metal materials. The results obtained through the use of a finite element simulation model showed that the fundamental amplitude is significantly reduced due to offset effects, while the second harmonic amplitude is increased due to superposition, thus increasing the *β* value to a significant extent.

Fatigued specimens of 0Cr17Ni4Cu4Nb martensitic stainless steel were tested. The experimental results conform to the simulated results. The method of exciting reversed-phase Rayleigh waves in opposite directions greatly increases *β*. The synchronous excitation of two angle beam wedge transducers could double the detection efficiency, which provides a new means for rapid and accurate detection of the fatigue damage in such materials. This approach has potential application prospects in engineering practice for the detection of surface micro-defects and the prediction of the fatigue life of thick-plate structures.

## Figures and Tables

**Table 1:** Simulation results of *A*_1_, *A*_2_, and *β* when exciting Rayleigh waves in different modes on the premise of having changing sizes and locations of micro-cracks.

Setting of Size and Location of Micro-Cracks	Excitation Mode	*A*_1_ (μm)	*A*_2_ (μm)	*β*
20 nm × 150 μm (−10.5,5) 20 nm × 150 μm (10,5)	P_1_	3.74 × 10^−2^	5.89 × 10^−3^	4.21 × 10^3^
	P_2_	3.41 × 10^−2^	1.71 × 10^−3^	1.47 × 10^3^
	P_1_ and P_2_	4.42 × 10^−3^	7.53 × 10^−3^	3.85 × 10^5^
20 nm × 200 μm (−10,5) 20 nm × 150 μm (10,5)	P_1_	3.75 × 10^−2^	5.61 × 10^−3^	3.99 × 10^3^
	P_2_	4.02 × 10^−2^	5.30 × 10^−3^	3.28 × 10^3^
	P_1_ and P_2_	4.66 × 10^−3^	1.08 × 10^−2^	4.97 × 10^5^
20 nm × 200 μm (−10.5,5) 20 nm × 150 μm(10,5)	P_1_	3.52 × 10^−2^	5.58 × 10^−3^	4.50 × 10^3^
	P_2_	3.39 × 10^−2^	8.18 × 10^−4^	7.12 × 10^2^
	P_1_ and P_2_	1.79 × 10^−3^	6.25 × 10^−3^	1.95 × 10^6^

**Table 2:** Values of *A*_1_, *A*_2_, and *β* of the specimen after 1.8 × 10^5^ fatigue cycles when exciting Rayleigh waves in different modes.

Excitation Mode	*A*_1_ (V)	*A*_2_ (V)	*β*
Only opening Channel 1	6.43	1.19 × 10^−2^	2.88 × 10^−4^
Only opening Channel 2	6.20	1.87 × 10^−2^	4.86 × 10^−4^
Synchronously opening Channels 1 and 2	1.77	2.28 × 10^−2^	7.27 × 10^−3^
